# Length of *FMR1* repeat alleles within the normal range does not substantially affect the risk of early menopause 

**DOI:** 10.1093/humrep/dew204

**Published:** 2016-09-17

**Authors:** Katherine S. Ruth, Claire E. Bennett, Minouk J. Schoemaker, Michael N. Weedon, Anthony J. Swerdlow, Anna Murray

**Affiliations:** 1 Genetics of Complex Traits, University of Exeter Medical School, RILD Level 3, Royal Devon & Exeter Hospital, Barrack Road, Exeter, EX2 5DW, UK; 2 Division of Genetics and Epidemiology, The Institute of Cancer Research, London, UK; 3 Division of Breast Cancer Research, The Institute of Cancer Research, London, UK

**Keywords:** MeSH, FMR1-related primary ovarian insufficiency, Fragile X-associated primary ovarian insufficiency, FMR1 protein, human, menopause, premature menopause

## Abstract

**STUDY QUESTION:**

Is the length of *FMR1* repeat alleles within the normal range associated with the risk of early menopause?

**SUMMARY ANSWER:**

The length of repeat alleles within the normal range does not substantially affect risk of early menopause.

**WHAT IS KNOWN ALREADY:**

There is a strong, well-established relationship between length of premutation *FMR1* alleles and age at menopause, suggesting that this relationship could continue into the normal range. Within the normal range, there is conflicting evidence; differences in ovarian reserve have been identified with *FMR1* repeat allele length, but a recent population-based study did not find any association with age at menopause as a quantitative trait.

**STUDY DESIGN, SIZE, DURATION:**

We analysed cross-sectional baseline survey data collected at recruitment from 2004 to 2010 from a population-based, prospective epidemiological cohort study of >110 000 women to investigate whether repeat allele length was associated with early menopause.

**PARTICIPANTS/MATERIALS, SETTING, METHOD:**

We included 4333 women from the Breakthrough Generations Study (BGS), of whom 2118 were early menopause cases (menopause under 46 years) and 2215 were controls. We analysed the relationship between length of *FMR1* alleles and early menopause using logistic regression with allele length as continuous and categorical variables. We also conducted analyses with the outcome age at menopause as a quantitative trait as well as appropriate sensitivity and exploratory analyses.

**MAIN RESULTS AND THE ROLE OF CHANCE:**

There was no association of the shorter or longer *FMR1* allele or their combined genotype with the clinically relevant end point of early menopause in our main analysis. Likewise, there were no associations with age at menopause as a quantitative trait in our secondary analysis.

**LIMITATIONS, REASONS FOR CAUTION:**

Women with homozygous alleles in the normal range may have undetected *FMR1* premutation alleles, although there was no evidence to suggest this. We estimate minor dilution of risk of early menopause from the likely inclusion of some women with menopause at over 45 years in the early menopause cases due to age-rounding bias in self-reports.

**WIDER IMPLICATIONS OF THE FINDINGS:**

There is no robust evidence in this large study that variation within the normal range of *FMR1* repeat alleles influences timing of menopause in the general population, which contradicts findings from some earlier, mainly smaller studies. The *FMR1* CGG repeat polymorphism in the normal range is unlikely to contribute to genetic susceptibility to early menopause.

**STUDY FUNDING/COMPETING INTEREST(S):**

We thank Breast Cancer Now and The Institute of Cancer Research for funding the BGS. The Institute of Cancer Research acknowledges NHS funding to the NIHR Biomedical Research Centre. The study was funded by the Wellcome Trust (grant number 085943). There are no competing interests.

**TRIAL REGISTRATION NUMBER:**

Not applicable.

## Introduction

Previous studies have suggested that normal variation in number of CGG repeats in *FMR1* could influence age at menopause. The 5′ untranslated region of the *FMR1* gene contains a CGG repeat that varies in length, causing Fragile X syndrome at over 200 repeats, with methylation and silencing of the *FMR1* gene and lack of FMRP expression. While ovarian function remains normal in women with full mutation range repeat alleles, primary ovarian insufficiency (POI) occurs in 20% of women with alleles in the premutation range of *FMR1* (55–200 repeats) (Allen *et al*., 2014).

In the premutation range, FMRP is expressed although there are elevated levels of *FMR1* mRNA, which have been found to sequester mRNA binding proteins. Although the mechanism of causation for POI remains unknown, premutation range alleles impair follicle development and induce apoptosis in mouse models (Lu *et al*., 2012). Also, there is a non-linear relationship between length of premutation alleles that have a dominant genetic effect and age at menopause, with earliest menopause at around 80 copies and later menopause at lower and higher copy numbers (Sullivan *et al*., 2005; Ennis *et al*., 2006; Mailick *et al*., 2014). It has been hypothesised that this relationship with age at menopause may continue to be observed in the range for normal length alleles (<55 repeats).

In white Europeans, the lowest observed allele length is six CGGs, and there are peaks in the distribution at 20, 23 and 30 CGGs, with 30 repeats being the most common (Murray *et al*., 1996). Previous studies have defined subgroups based on allele length and have reported differences in ovarian reserve between these (Gleicher *et al*., 2009, 2010, 2012a,b). In some studies, a greater proportion of longer normal length *FMR1* alleles have been found in women with POI or diminished ovarian reserve (Bretherick *et al*., 2005; Bodega *et al*., 2006; Pastore *et al*., 2012). However, many of these studies were composed of fewer than 500 women. Other analyses, including two large studies in over 3000 women by Voorhuis *et al*., have not found an association between longer length normal *FMR1* alleles and POI (Bennett *et al*., 2010; Voorhuis *et al*., 2014) and no relationship between normal length *FMR1* alleles and age at menopause (Voorhuis *et al*., 2013). We tested the role of normal-sized *FMR1* CGG repeat alleles in menopause timing in a cohort of over 2000 early menopause cases, plus over 2000 controls, drawn from a population-based study of over 110 000 women from the Breakthrough Generations Study (BGS).

## Materials and Methods

### Participants included

In this analysis, we included 4333 women from the BGS recruited from 2004 to 2010. The BGS is a prospective population cohort study started in 2003 to investigate the environmental, behavioural, hormonal and genetic causes of breast cancer (Swerdlow *et al*., 2011) (http://www.breakthroughgenerations.org.uk/). The BGS cohort includes over 110 000 women aged 16 and older at entry, recruited from the general UK population through connections to the charity Breakthrough Breast Cancer or who volunteered as a result of publicity, and female friends and family members of these participants. The study received appropriate ethical approval from the South East MREC, and informed consent was received from the participants (Swerdlow *et al*., 2011). Detailed menstrual histories and blood samples were collected. All women with early menopause were eligible for inclusion in our analyses. Breast cancer cases were excluded from our analyses. Of the women included, 99.5% were of white ethnicity (Table I).

**Table I:** Summary statistics for individuals included in the analysis (all variables measured at recruitment unless otherwise specified).

	Early menopause cases		Controls		Post-menopausal women included in quantitative trait analysis	
Age (years)	*n* = 2118		*n* = 2215		*n* = 3805	
Mean (range)	58.5 (22,88)		59.4 (45,89)		60.3 (22,89)	
Median (lower quartile, upper quartile)	58 (52,64)		59 (53,65)		60 (55,66)	
Year of entry	*n* = 2118		*n* = 2215		*n* = 3805	
Mean (range)	2006 (2004, 2009)		2006 (2004, 2010)		2006 (2004, 2010)	
Median (lower quartile, upper quartile)	2006 (2005, 2007)		2006 (2005, 2007)		2006 (2005, 2007)	
Age at menopause	*n* = 2118		*n* = 1687		*n* = 3805	
Mean (range)	42.5 (19,45)		52.1 (46,62)		46.7 (19,62)	
Median (lower quartile, upper quartile)	43 (41,45)		52 (50,54)		45 (43,52)	
BMI at age 20	*n* = 1684		*n* = 1801		*n* = 3053	
Mean (range)	21.3 (12,56.5)		21.4 (12.4,38.3)		21.3 (12,56.5)	
Median (lower quartile, upper quartile)	21 (19.6,22.6)		21.1 (19.8,22.7)		21 (19.7,22.6)	
BMI (kg/m^2^)	*n* = 2041		*n* = 2148		*n* = 3676	
Mean (range)	25.8 (5.1,63.2)		25.6 (14.9,54.4)		25.7 (5.1,63.2)	
Median (lower quartile, upper quartile)	24.9 (22.7,28)		24.9 (22.6,27.6)		24.9 (22.7,27.8)	
Height (m)	*n* = 2082		*n* = 2192		*n* = 3750	
Mean (range)	1.63 (1.27,2.06)		1.63 (1.37,1.83)		1.63 (1.27,2.06)	
Median (lower quartile, upper quartile)	1.63 (1.58,1.68)		1.63 (160,1.68)		1.63 (1.58,1.68)	
Number of births (live and still) at ≥26 weeks gestation (includes never pregnant)	*n* = 2111		*n* = 2210		*n* = 3795	
Mean (range)	1.9 (0,10)		2.1 (0,8)		2.0 (0,10)	
Median (lower quartile, upper quartile)	2 (1,3)		2 (2,3)		2 (1,3)	
	***n***	***%***	***n***	***%***	***n***	***%***
Ethnicity						
White ethnicity	2107	99.5	2204	99.5	3787	99.5
Non-white	11	0.5	11	0.5	18	0.5
Smoking status						
Never smoker	1242	58.6	1401	63.3	2299	60.4
Former smoker	683	32.3	714	32.2	1146	32.8
Current smoker	187	8.8	99	4.5	252	6.7
Smoking status not known	6	0.3	1	0.1	7	0.2
Hormone replacement therapy use						
Never	1036	48.9	1526	68.9	2077	54.6
Former	752	35.5	539	24.3	1268	33.3
Current	323	15.3	147	6.6	450	11.8
Not known	7	0.3	3	0.1	10	0.3
Oral contraceptive use						
Never	526	24.8	584	26.4	1065	28.0
Former	1569	74.1	1591	71.8	2711	71.3
Current	11	0.5	35	1.6	12	0.3
Not known	12	0.6	5	0.2	17	0.4
Total	2118		2215		3805	

### Definition of age at natural menopause

Natural menopause was defined as cessation of menstruation for at least 6 months without known cause based on questionnaire data (‘Have your periods now stopped completely? (That is, have you now gone at least 6 months without having a period and you are not pregnant or on the contraceptive pill)’). We excluded women if periods stopped because of pregnancy, breastfeeding, surgery, hormonal contraceptive use or other types of medical treatment or if there was a medical condition or illness that could have caused amenorrhoea (e.g. polycystic ovary syndrome).

### Early menopause cases and controls

A nested case–control design was used for reasons of cost-effectiveness. Early menopause cases had natural menopause at age 45 years or younger, whereas controls were women known to have had menopause (natural or surgical) at 46 years or older or, to allow sufficient suitable controls to be identified, women who were aged 46 years or older and were premenopausal. Where possible, controls were matched to cases on date of birth (within 12 months), ethnicity, year of questionnaire completion and source of recruitment. Where this was not possible, matching criteria were relaxed in the following order until a match was found: source of recruitment, year of completion and date of birth. Early menopause cases aged 45 years or younger were matched to a control aged 46 years, since all postmenopausal women aged 45 years or younger were early menopause cases. In order to exclude subjects who were genetically related to each other, we identified mother–daughter and sister–sister pairs among the selected subjects. We excluded the oldest subject when both of the individuals were cases or controls. When one was a case and the other a control, the control was replaced. Following genotyping and data cleaning, 4333 women remained in our analysis, of whom 2215 were early menopause cases (including 250 women with menopause under 40 years) and 2118 were controls.

### Evaluation of *FMR1* repeat length

For each subject, Asuragen Amplidex kits (http://www.asuragen.com) containing *FMR1* CGG repeat region–specific primers were used to PCR amplify the *FMR1* repeat region from 20 ng of genomic DNA that had been extracted from peripheral blood mononuclear cells. All PCRs were performed in 3 μl of reaction volumes in 384-well microtiter plates, using conditions recommended by the kit manufacturers. Products were size separated by capillary electrophoresis on an ABI 3730 automated sequencer (Applied Biosystems, Warrington, UK), using a ROX 1000 size standard (Asuragen, Austin, TX, USA) for estimation of product sizes. CGG repeat numbers were determined by comparison with a control individual of 52 CGG repeats. We included duplicates of ~10% of the samples on independent plates. The concordance between duplicate samples was 98.5%, excluding differences of ±1 CGG repeat. Controls included 12 no-template controls, 3 samples from females of known expansion size (largest CGG = 55, 117 and 145) and a lane containing the multiple size targets supplied by Asuragen (CGG = 20, 29, 31, 53, 117 and 196) per 384 plate.

### Statistical analyses

We analysed the effect of *FMR1* alleles and genotypes on odds of early menopause using logistic regression. All women had both *FMR1* repeat alleles in the clinically normal range (<55 copies). All statistical analyses were performed using Stata 13.1 or 14.1 (StataCorp, College Station, TX, USA). To avoid assuming that normal length *FMR1* alleles would act by the same dominant genetic mechanism as premutation alleles, we considered dominant and additive effects by investigating the effects of each individual *FMR1* allele and combinations of alleles (genotypes).

In each woman, we defined ‘Allele 1’ as the shorter *FMR1* repeat allele and ‘Allele 2’ as the longer of her two alleles. We tested the per repeat effect of each *FMR1* allele as a continuous variable and also classified each allele into repeat categories according to size, using previously published criteria (Gleicher *et al*., 2014): alleles <26 repeats were considered to be ‘low’, 26–34 repeats to be ‘medium’ and >34 repeats to be ‘high’.

To test the effects of each individual *FMR1* allele, statistical analyses were performed for Allele 1 and Allele 2 separately. Allele length was treated in three ways: as a continuous variable, as a nominal categorical variable (comparison of low and high to medium length as the reference category) or as an ordinal categorical variable (analysed as a continuous variable ordered from low to high).

We also analysed the effect of the *FMR1* repeat allele combination, or genotype. We included each allele as a continuous variable in the same model, with/without an interaction term (Allele 1 × Allele 2). We generated six genotype categories from the combinations of repeat length category for the two alleles: low/low, low/medium, low/high, medium/medium, medium/high, high/high. The genotypes were treated as either nominal variables (comparing each genotype to medium/medium) or as ordinal variables (analysed as a continuous variable), defining the order in two ways: (i) (a) low/low, (b) low/medium, (c) low/high, (d) medium/medium, (e) medium/high, (f) high/high; or (ii) (a) low/low, (b) low/medium, (c) medium/medium, (d) low/high, (e) medium/high, (f) high/high. Exploratory analyses were performed of alternative methods of modelling combinations of the alleles: as a difference between allele length in an individual or as a mean allele length.

In a secondary analysis, we investigated the relationship between *FMR1* alleles and genotypes with age at natural menopause in the 3805 postmenopausal women in our study. This was a smaller number than in the case–control study because not all controls were postmenopausal and so some were not included. The residuals of the regression on menopause age were not normally distributed due to overrepresentation of early menopause cases in the analysis; therefore, we applied a statistical correction to transform the data to a normal distribution. Briefly, this method ranked the observed values of age at menopause (randomly ordering tied values), then converted these to a percentile of a standard normal distribution.

Sensitivity analyses were carried out by conditional logistic regression analyses in 1559 fully matched case–control pairs, and by repeating the logistic regression analyses restricted to women of white ethnicity and including smoking status at time of study entry as a covariate. Smoking status and ethnicity were available for all women analysed (Table I). In the analysis of menopause as a quantitative trait, we also tested the models separately in the control group. Earlier exploratory analysis with menopause before 40 years as the outcome was consistent with the main analysis (not presented).

Statistical power was estimated using Quanto (http://biostats.usc.edu/Quanto.html). We estimated the size of genetic effect we could detect at 80% power for an additive mode of inheritance comparing the low/low genotype with low/medium and medium/medium. For case–control models, we assumed a population prevalence of 5% for early menopause.

## Results

### Repeat distribution

**Figure 1:**
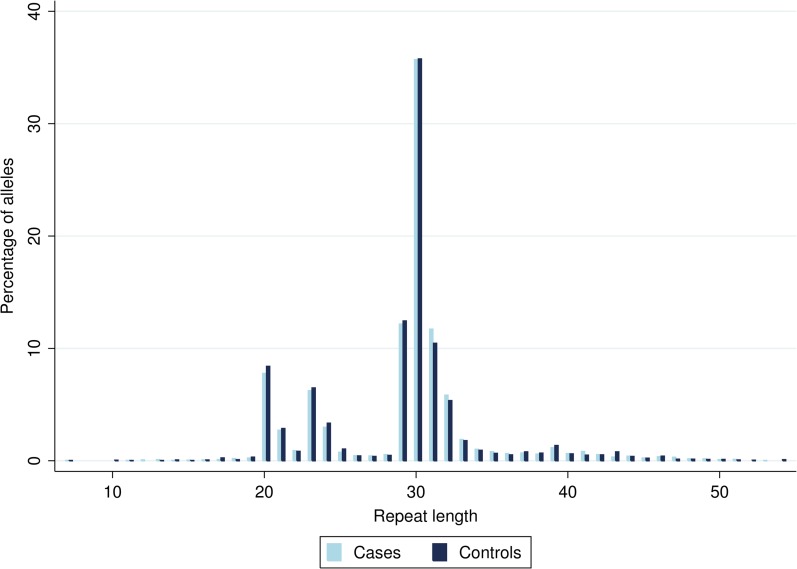
Percentage of *FMR1* alleles of each length in early menopause cases (*n* = 4236) and controls (*n* = 4430).

The length of the *FMR1* allele repeat ranged from 7 to 54 copies (Fig. 1). The distribution of the *FMR1* allele was consistent with previous studies with a mode at 30 copies, and secondary peaks at 20 and 23 copies (Fu *et al*., 1991; Peprah, 2012), with similar distributions in the early menopause cases and the rest of the cohort. Almost half of the women with a natural age at menopause had two medium length alleles (26–34 repeats), and almost one-third had a combination of one low allele (< 26 repeats) and one medium allele (26–34 repeats) (Table II).

**Table II:** Number of women by *FMR1* genotype, categorised by allele lengths.

Genotype	Early menopause cases		Controls		Postmenopausal women included in quantitative trait analysis	
	*n*	%	*n*	%	*n*	%
Low/low	130	5.9	101	4.8	204	5.4
Low/medium	710	32.1	662	31.3	1215	31.9
Low/high	88	4.0	77	3.6	145	3.8
Medium/medium	1027	46.4	1035	48.9	1803	47.4
Medium/high	248	11.2	221	10.4	409	10.7
High/high	12	0.5	22	1.0	29	0.8
Total	2215	100.0	2118	100.0	3805	100.0

Note: Low < 26 repeats; medium 26–34 repeats; high 35–54 repeats.

### Age of menopause

In early menopause cases (*n* = 2118), the median age at natural menopause was 43 years (range 19–45 years, mean 42.5 years, SD 3.1 years) compared with 52 years in the controls (*n* = 1687, range 46−62 years, mean 52.1 years, SD 3.1 years), although 24% (*n* = 528) of controls were premenopausal and therefore could not be included in the mean (Table I).

### No association between length of either *FMR1* repeat allele and early menopause

We found no associations for either *FMR1* allele with odds of early menopause (Table III). There was no association between length of either Allele 1 or Allele 2 of *FMR1* and age at menopause as a quantitative trait (*P >* 0.05 for all) (Table III).

**Table III:** Relationship of *FMR1* allele length with early menopause and age at menopause as a quantitative trait.

Model	Variables included	Early menopause (*n* = 2215 cases, *n* = 2118 controls)			Age at natural menopause (*n* = 3805)		
		OR	95% CI	*P*	Effect^a^	95% CI	*P*
Allele 1, continuous	Allele 1 (continuous)	1.01	1–1.03	0.077	0.00	−0.01–0	0.280
Allele 1, nominal categorical	1. Low	0.92	0.81–1.04	0.173	0.05	−0.01–0.12	0.125
	2. Medium	ref.			ref.		
	3. High	1.86	0.92–3.78	0.085	−0.26	−0.63–0.1	0.159
Allele 1, ordinal categorical	1. Low	1.11	0.99–1.25	0.075	−0.06	−0.12–0	0.063
	2. Medium						
	3. High						
Allele 2, continuous	Allele 2 (continuous)	1.00	0.99–1.01	0.750	0.00	−0.01–0.01	0.672
Allele 2, nominal categorical	1. Low	0.80	0.61–1.04	0.094	0.05	−0.07–0.22	0.302
	2. Medium	ref.			ref.		
	3. High	0.94	0.8–1.11	0.474	0.01	−0.07–0.11	0.679
Allele 2, ordinal categorical	1. Low	1.03	0.9–1.17	0.712	−0.01	−0.08–0.06	0.800
	2. Medium						
	3. High						

^a^Effect size is in standard deviations of inverse normally transformed age at menopause.

Notes: OR, odds ratio; ref., reference category.

### No association between *FMR1* repeat genotype and early menopause

*FMR1* repeat genotype was not associated with early menopause or age at menopause as a quantitative trait (*P* > 0.05 for all) (Table IV).

**Table IV:** Relationship of *FMR1* genotype with early menopause and age at menopause as a quantitative trait.

Model	Variables included	Early menopause (*n* = 2215 cases, *n* = 2118 controls)			Age at natural menopause (*n* = 3805)		
		OR	95% CI	*P*	Effect^a^	95% CI	*P*
Allele 1, Allele 2, continuous	Allele 1 (continuous)	1.01	1–1.03	0.079	0.00	−0.01–0	0.328
	Allele 2 (continuous)	1.00	0.98–1.01	0.805	0.00	−0.01–0.01	0.808
Allele 1, Allele 2 and interaction, continuous	Allele 1 (continuous)	0.98	0.9–1.06	0.586	0.00	−0.04–0.04	0.934
	Allele 2 (continuous)	0.97	0.91–1.04	0.367	0.00	−0.03–0.04	0.836
	Allele 1 × Allele 2 (interaction)	1.00	1–1	0.384	0.00	0–0	0.793
Genotype, nominal categorical	1. Low/low	0.77	0.59–1.01	0.063	0.09	−0.05–0.24	0.202
	2. Low/medium	0.93	0.81–1.06	0.265	0.06	−0.02–0.13	0.127
	3. Low/high	0.87	0.63–1.19	0.384	0.04	−0.13–0.21	0.619
	4. Medium/medium	ref.			ref.		
	5. Medium/high	0.88	0.72–1.08	0.230	0.05	−0.06–0.16	0.349
	6. High/high	1.82	0.9–3.7	0.098	−0.25	−0.61–0.12	0.190
Genotype, ordinal categorical (order 1)	1. Low/low	1.04	0.99–1.09	0.132	−0.02	−0.05–0	0.110
	2. Low/medium						
	3. Low/high						
	4. Medium/medium						
	5. Medium/high						
	6. High/high						
Genotype, ordinal categorical (order 2)	1. Low/low	1.03	0.97–1.09	0.399	−0.02	−0.05–0.01	0.309
	2. Low/medium						
	3 .Medium/medium						
	4. Low/high						
	5. Medium/high						
	6. High/high						

OR, odds ratio; ref., reference category.

^a^Effect size is in standard deviations of inverse normally transformed age at menopause.

Notes: The genotypes were treated as either nominal variables (comparing each genotype to medium/medium) or as ordinal variables, defining the order in two ways: (i) 1. low/low, 2. low/medium, 3. low/high, 4. medium/medium, 5. medium/high, 6. high/high; (ii) 1. low/low, 2. low/medium, 3. medium/medium, 4. low/high, 5. medium/high, 6. high/high.

### Sensitivity analyses

In the fully matched case–control analysis, we found no robust associations of either *FMR1* allele or genotype with odds of early menopause, although ‘low’ lengths of Allele 2 (OR = 0.72, 95% CI 0.52−0.99, *P* = 0.044) and the ‘low/low’ genotype (OR = 0.69; 95% CI 0.50–0.96; *P* = 0.028) were associated with decreased odds of early menopause (*P* > 0.05 for all other results) (Supplementary Table 1 and 2).

We identified smoking as a potential confounder, since smoking is associated with earlier menopause (Gold, 2011;Morris *et al*., 2012). Consistent with this, there was an association between being a current smoker and earlier menopause in our analyses (OR = 2.13; 95% CI 1.65–2.75; *P* = 6.4 × 10^–9^); however, length of *FMR1* allele or genotype was not associated with smoking (*P* > 0.05 for all) (data available on request). When the analyses were adjusted for smoking, the results were consistent with the main analysis (data available on request). The results remained consistent with the main analysis when analyses were restricted to women of white ethnicity (data available on request) or when the secondary analysis of age at menopause was carried out in only the control group (Supplementary Tables 3 and 4). In exploratory analyses, neither mean repeat length nor difference in repeat length was associated with early menopause or age at menopause (*P* > 0.05 for all).

## Discussion

We found no robust association between normal length *FMR1* alleles and early menopause. This is unlikely to be due to a lack of power since we estimated that we were powered to detect an odds ratio <0.85 or >1.18 per low allele (<26 repeats) in the analysis of all cases and controls (with similar values for matched analysis). This is similar in size to estimated odds ratios for early menopause (≤45 years) of 1.13–1.85 per allele for common single-nucleotide polymorphisms (SNPs) in the same study cohort (Murray *et al*., 2011). For the analysis of age at menopause as a quantitative trait, we estimated that we were powered to detect a change of about 0.5 years per low allele, similar to the 0.1–0.9 years per allele effect sizes for common SNPs (Day *et al*., 2015). Indeed, we were sufficiently powered to detect a strong effect of smoking on reducing age of menopause, which has been previously observed in this same study cohort (Morris *et al*., 2012).

As well as demonstrating no association between normal *FMR1* allele length and risk of early menopause, our results corroborate a null association between *FMR1* normal length alleles and quantitative age at menopause from a population-based study not focussed on early menopause (Voorhuis *et al*., 2013). Accelerated loss of ovarian reserve in women with low alleles and better ovarian reserve with high alleles has been reported in studies including up to 521 women (Gleicher *et al*., 2010, 2012a,b, 2014). Our study is not consistent with these findings since lower AMH levels, and hence ovarian reserve, predict earlier menopause (Aydogan and Mirkin, 2015); however, reduced ovarian reserve does not necessarily result in POI and this may explain the discrepancy (Gleicher *et al*., 2015).

Although we were well-powered to detect an association, our calculations do not take into account factors affecting the accuracy of the data collected or that would have reduced our power to detect an association. Long *FMR1* alleles are harder to detect; therefore, women with homozygous alleles may actually be heterozygotes with an undetected premutation repeat. Of the 4333 women, 21% were homozygotes, but there was high concordance between duplicated samples, and the proportion of homozygotes in early menopause cases and controls was not statistically different. We would expect such genotyping errors to result in a higher proportion of homozygotes in early menopause cases, since premutation repeats are a known aetiological risk factor for early menopause (Sherman, 2000).

Another factor that might have contributed to the lack of an observed association is the potential dilution of risk of early menopause from misidentification of early menopause cases. Previous studies have observed rounding bias towards reporting values ending in 0 or 5 when women are asked to recall their age at menopause (Hahn *et al*., 1997); therefore, some women may have rounded down their menopause age to 45. We estimate, based on the distribution in our data by reported age in single years, that this may have occurred in 7% of early menopause cases. Hence, the consequent dilution of risk would have been minor and does not account for the lack of an observed association. We controlled for two potential confounders in our analysis: ethnicity and smoking. Ethnicity is known to affect *FMR1* allele length (Peprah, 2012), and we found no association with smoking.

In summary, in a large population-based study, we found no association between normal length *FMR1* repeats and risk of the clinically relevant outcome early menopause, and replicated a null association with age at menopause as a quantitative trait. The *FMR1* CGG repeat polymorphism in the normal range does not influence risk of early menopause and is therefore unlikely to contribute to genetic susceptibility to early menopause.

## Supplementary data

Supplementary data are available at http://humrep.oxfordjournals.org/.

Supplementary Data
