# Supplementary material for: Length of FMR1 repeat alleles within the normal range does not substantially affect the risk of early menopause
Source: Hum Reprod. 2016 Sep 17;31(10):2396–403. doi: 10.1093/humrep/dew204 (PMC5027929; doi:10.1093/humrep/dew204)
Supplement: Supplementary Data [file supp_31_10_2396__index.html]

Length of FMR1 repeat alleles within the normal range does not substantially affect the risk of early menopause — Supplementary Data 

# Length of *FMR1* repeat alleles within the normal range does not substantially affect the risk of early menopause

## Supplementary Data

Supplementary Data

- Supplementary Data - pdf file
- Supplementary Data - pdf file
- Supplementary Data - pdf file
- Supplementary Data - pdf file
