# Supplementary material for: Length of FMR1 repeat alleles within the normal range does not substantially affect the risk of early menopause
Source: Hum Reprod. 2016 Sep 17;31(10):2396–403. doi: 10.1093/humrep/dew204 (PMC5027929; doi:10.1093/humrep/dew204)
Supplement: Supplementary Data [file supp_dew204_dew204supp_table1.pdf]

Human Reproduction, 2016
doi:10.1093/humrep/dew204

human reproduction

SUPPLEMENTARY DATA

Supplementary Table SI **Relationship of *FMR1* allele length with early menopause in matched cases and controls (*n* = 3118).**

Model	Variables included	OR	95% LCL	95% UCL	SE	*P*
Allele 1 (cont.)	Allele 1 (cont.)	1.012	0.997	1.028	0.008	0.116
Allele 1, categorical nominal	1. low	0.913	0.792	1.052	0.066	0.207
	2. medium (ref.)	ref.	ref.	ref.	ref.	ref.
	3. high	2.339	0.968	5.651	1.053	0.059
Allele 1, categorical ordinal	(1) low, (2) medium, (3) high	1.129	0.983	1.296	0.079	0.086
Allele 2 (cont.)	Allele 2 (cont.)	1.004	0.989	1.020	0.008	0.574
Allele 2, categorical nominal	1. low	0.721	0.524	0.991	0.117	**0.044**
	2. medium (ref.)	ref.	ref.	ref.	ref.	ref.
	3. high	0.963	0.790	1.174	0.097	0.712
Allele 2, categorical ordinal	(1) low, (2) medium, (3) high	1.075	0.917	1.261	0.087	0.372

The value in bold is significant at $P < 0.05$; cont, continuous; LCL, lower limit of 95% confidence interval; OR, odds ratio; ref., reference category; SE, standard error; UCL, upper limit of 95% confidence interval.
