# Supplementary material for: Length of FMR1 repeat alleles within the normal range does not substantially affect the risk of early menopause
Source: Hum Reprod. 2016 Sep 17;31(10):2396–403. doi: 10.1093/humrep/dew204 (PMC5027929; doi:10.1093/humrep/dew204)
Supplement: Supplementary Data [file supp_dew204_dew204supp_table2.pdf]

Supplementary Table SII **Relationship of *FMR1* genotype with early menopause in matched cases and controls ($n = 3118$).**

Model	Variables included	OR	95% LCL	95% UCL	SE	*P*
Allele 1 and Allele 2 (cont.)	Allele 1 (cont.)	1.012	0.996	1.029	0.009	0.143
	Allele 2 (cont.)	1.000	0.984	1.017	0.008	0.977
Allele 1, Allele 2 and interaction (cont.)	Allele 1 (cont.)	1.017	0.927	1.115	0.048	0.726
	Allele 2 (cont.)	1.004	0.930	1.083	0.039	0.926
	Allele 1 × Allele 2 (interaction)	1.000	0.997	1.003	0.001	0.929
Genotype, categorical nominal	1. low/low	0.695	0.502	0.961	0.115	**0.028**
	2. low/medium	0.915	0.778	1.075	0.075	0.279
	3. low/high	0.953	0.651	1.395	0.185	0.804
	4. medium/ medium (ref.)	ref.	ref.	ref.	ref.	ref.
	5. medium/high	0.861	0.677	1.095	0.105	0.222
	6. high/high	2.292	0.947	5.545	1.033	0.066
Genotype, categorical ordinal (order 1)	(1) low/low,<br>(2) low/medium,<br>(3) low/high,<br>(4) medium/medium (ref.),<br>(5) medium/high,<br>(6) high/high	1.049	0.990	1.112	0.031	0.104
Genotype, categorical ordinal (order 2)	(1) low/low,<br>(2) low/medium,<br>(3) medium/medium (ref.),<br>(4) low/high,<br>(5) medium/high,<br>(6) high/high	1.042	0.973	1.115	0.036	0.239

The value in bold is significant at $P < 0.05$.
