# Supplementary material for: Length of FMR1 repeat alleles within the normal range does not substantially affect the risk of early menopause
Source: Hum Reprod. 2016 Sep 17;31(10):2396–403. doi: 10.1093/humrep/dew204 (PMC5027929; doi:10.1093/humrep/dew204)
Supplement: Supplementary Data [file supp_dew204_dew204supp_table3.pdf]

Supplementary Table SIII **Relationship of *FMR1* allele length with age at menopause as a quantitative trait in controls (n = 1,687[a]).**

Model	Variables included	Beta[b]	95% LCL	95% UCL	SE	*P*
Allele 1 (cont.)	Allele 1 (cont.)	0.007	−0.004	0.018	0.006	0.207
Allele 1, categorical nominal	1. low	−0.028	−0.125	0.068	0.049	0.564
	2. medium (ref.)	ref.	ref.	ref.	ref.	ref.
	3. high	−0.009	−0.750	0.733	0.378	0.982
Allele 1, categorical ordinal	(1) low, (2) medium, (3) high	0.028	−0.067	0.122	0.048	0.569
Allele 2 (cont.)	Allele 2 (cont.)	−0.001	−0.011	0.009	0.005	0.810
Allele 2, categorical nominal	1. low	−0.090	−0.290	0.110	0.102	0.375
	2. medium (ref.)	ref.	ref.	ref.	ref.	ref.
	3. high	−0.032	−0.164	0.100	0.067	0.630
Allele 2, categorical ordinal	(1) low, (2) medium, (3) high	0.007	−0.097	0.111	0.053	0.894

[a]528 controls were pre-menopausal but aged ≥46 years and so were not included in this analysis.

[b]Effect size is in standard deviations of inverse-normally transformed age at menopause.
