# Supplementary material for: Length of FMR1 repeat alleles within the normal range does not substantially affect the risk of early menopause
Source: Hum Reprod. 2016 Sep 17;31(10):2396–403. doi: 10.1093/humrep/dew204 (PMC5027929; doi:10.1093/humrep/dew204)
Supplement: Supplementary Data [file supp_dew204_dew204supp_table4.pdf]

Supplementary Table SIV **Relationship of *FMR1* genotype with age at menopause as a quantitative trait in controls (n = 1687[a]).**

Model	Variables included	Beta [b]	95% LCL	95% UCL	SE	*P*
Allele 1 and Allele 2 (cont.)	Allele 1 (cont.)	0.008	−0.003	0.020	0.006	0.161
	Allele 2 (cont.)	−0.004	−0.014	0.007	0.005	0.513
Allele 1, Allele 2 and interaction (cont.)	Allele 1 (cont.)	0.019	−0.049	0.087	0.035	0.590
	Allele 2 (cont.)	0.005	−0.051	0.061	0.029	0.860
	Allele 1 × Allele 2 (interaction)	0.000	−0.002	0.002	0.001	0.760
Genotype, categorical nominal	1. low/low	−0.102	−0.307	0.103	0.105	0.330
	2. low/medium	−0.028	−0.137	0.082	0.056	0.621
	3. low/high	−0.029	−0.277	0.219	0.126	0.819
	4. medium/medium (ref.)	ref.	ref.	ref.	ref.	ref.
	5. medium/high	−0.050	−0.209	0.109	0.081	0.536
	6. high/high	−0.018	−0.761	0.724	0.379	0.961
Genotype, categorical ordinal (order 1)	(1) low/low,<br>(2) low/medium,<br>(3) low/high,<br>(4) medium/medium (ref.),<br>(5) medium/high,<br>(6) high/high	0.011	−0.029	0.050	0.020	0.600
Genotype, categorical ordinal (order 2)	(1) low/low,<br>(2) low/medium,<br>(3) medium/medium (ref.),<br>(4) low/high,<br>(5) medium/high,<br>(6) high/high	0.005	−0.041	0.051	0.024	0.841

[a]528 controls were pre-menopausal but aged ≥46 years and so were not included in this analysis.

[b]Effect size is in standard deviations of inverse-normally transformed age at menopause.
